# Cathepsin D in prawn reproductive system: its localization and function in actin degradation

**DOI:** 10.7717/peerj.10218

**Published:** 2020-11-11

**Authors:** Chompoonut Sukonset, Piyaporn Surinlert, Orawan Thongsum, Atthaboon Watthammawut, Monsicha Somrit, Jirasuda Nakeim, Wattana Weerachatyanukul, Somluk Asuvapongpatana

**Affiliations:** 1 Department of Anatomy, Faculty of Science, Mahidol University, Ratchathewee, Bangkok, Thailand; 2 Chulabhon International College of Medicine, Thammasat University, Prathumtani, Pratumtani, Thailand; 3 Department of Anatomy, Faculty of Medicine, Srinakharinwirot University, Wattana, Bangkok, Thailand; 4 Department of Anatomy, Faculty of Allied Health Science, Buraphar University, Mueng Chonburi, Chonburi, Thailand

**Keywords:** CAT-D, Testis, Sperm, Spermatogenesis, Actin meshwork

## Abstract

Cathepsin D (CAT-D) is a well-known aspartic protease that serves a function as house-keeping lysosomal enzyme in all somatic cells. Its existence in reproductive tissues is highly variable, even in the somatic derived epithelial cells of reproductive tract. In *Macrobrachium rosenbergii*, existence of MrCAT-D and its translational product was detected in both somatic cells (Sertoli-like supporting cells) and developing spermatogenic cells as well as along accessory spermatic ducts. Specifically, MrCAT-D was localized onto the sperm surface rather than within the acrosomal matrix, as evident by similar staining pattern of anti-CAT-D on live and aldehyde fixed sperm. MrCAT-D in testicular extracts and sperm isolates showed active enzyme activities towards its specific fluorogenic substrate (MCA-Gly-Lys-Pro-Ile-Leu-Phe-Phe-Arg-Leu-Lys (Dnp)-D-Arg-NH_2_). MrCAT-D also exerted its function towards hydrolyzing filamentous actin, the meshwork of which is shown to be localized at the junction between germ cells and supporting cells and spermatogonia in *M. rosenbergii* testicular epithelium. Together, we have localized MrCAT-D transcript and its translational product in both supporting and germ cells of testis and claimed its enzymatic function towards actin degradation, which may be related to sperm release from the epithelial cell interaction.

## Introduction

Sperm enzymes have gained a lot of attention in two aspects based on their residence: one is the enzymes residing in the acrosomal sac while the others are those anchoring or adhering on the sperm surface. In mammals, several subtypes of the enzymes including serine proteases (Polakoski, McRorie & Williams, 1973; Honda, Siruntawineti & Baba, 2002), metalloproteases (Ferrer et al., 2012; Yudin et al., 1999), proteasomes (Sutovsky et al., 2004; Yi et al., 2009; Miles & Sutovsky, 2014) and many more falls into the former category who play their essential role in egg vestment digestion to create a path for sperm entry into egg proper. The latter category or sperm surface enzymes are also accumulatively documented such as mannosidases, arylsulfatases and many other glycosidases (Cornwall, Tulsiani & Orgebin-Crist, 1991; Tulsiani et al., 1995; Weerachatyanukul et al., 2003). These sperm enzymes could be either sperm-inherent or fluid-absorbed proteins (secreted from epididymal origin) which serve two main functions: (1) they presumably hydrolyze the bridges between testicular sperm and Sertoli cells during spermiation (De Kretser & Kerr, 1994; Yanagimachi, 1994) and (2) they serve as ligands to interact with egg receptors during gamete interaction (Wassarman & Litscher, 2000; Carmona et al., 2002). In crustaceans and other aquatic animals, the existence and properties of many enzymes in digestive and immune systems have been gradually accumulated (Garcia-Carreño, Navarrete del Toro & Muhlia-Almazan, 2014; Bañuelos Vargas et al., 2018). In the reproductive system, presence of a trypsin-like protease as sperm surface enzymes and its activation in association with post-testicular sperm modifications prior to gamete interaction have been reported in shrimp and prawn species (Suphamungmee et al., 2008; Watthammawut et al., 2015). Here, we further characterized the other type of sperm surface enzyme, cathepsin-D (CAT-D), and demonstrated its role in actin filament hydrolysis.

Cathepsin-D (CAT-D) is a well characterized aspartic enzyme found in most somatic cells while it has much been less studied in reproductive system. As it falls in the aspartic enzyme subgroup, the unique three “catalytic box” amino acids, Asp-Thr-Glu, is well conserved among these enzymes. For substrate interaction, CAT-D requires hydrophobic amino acid cluster at the cleavage site (P1 and P1′ positions) and substrate preference P2 site, whereas, the polar amino acid is of preference at the P2′ position (Pimenta et al., 2001). In the reproductive system, we have reported its existence in mammalian male reproductive tissues and its surface localization which is acquired from its adsorption from epididymal fluid as part of sperm maturation process (Asuvapongpatana et al., 2013; Saewu et al., 2012). In a few original works of mammalian reproductions, the unique localization of CAT-D as a cell- or region-specific fashion has been revealed in testis and epididymis (Igdoura, Morales & Hermo, 1995; Hermo & Andonian, 2003), despite the fact that CAT-D is abundantly detected in the lysosomes of all somatic cells (Benes, Vetvicka & Fusek, 2008; Masson et al., 2010). In testis, CAT-D is specifically localized in lysosomes of Sertoli cells, while it has not been detected in spermatogenic cells, particularly acrosome which is physiologically homologous to lysosomes (Huo et al., 2008; Asuvapongpatana et al., 2013; Saewu et al., 2012). It has been proposed functionally that hydrolytic proteases in Sertoli cell cytoplasm such as cathepsins and other serine proteases exert their function to cleave integrin-ECM interaction to release testicular sperm into the adluminal compartment (Yanagimachi, 1994; Cheng & Mruk, 2011) . Two types of junctional-like complexes existing along surface of Sertoli cells are “ectopic specialization” (ES) and “tubulobulbar complex” (TBC) which are made up of filamentous actin meshwork (O’Donnell et al., 2011; Vogl et al., 2014). Hydrolysis and disruption of this actin meshwork is thus directly associated with the release of testicular sperm from their interacted epithelium (Yanagimachi, 1994; Cheng & Mruk, 2011). As filamentous actin is one of the physiological substrates of CAT-D (Hughes et al., 2000), it is reasonable to proposal in this study that surface MrCAT-D may serve a function to hydrolyze actin meshwork that is known to localize in-between late spermatids and supporting cells, a similar spermiation function as that reported in mammals mentioned above.

## Materials & Methods

### Prawns and sperm collection

Three-month old, blue-claw giant freshwater prawns with an average weight of 125 g were purchased from the Central Market for the Promotion of Agriculture in Ayutthaya province, Thailand. The acquired prawns were transported and reared prior to sample extraction according to the guidelines of the National Research Council of Thailand, the National Aquaculture Council of Australia (Johnston & Jungalwalla, 2005), Seafish UK (Jacklin & Combes, 2007) and the Animal Care Committee, Mahidol University, Thailand (MUSC-IACUC, protocol # 2016/014). Immediately prior to sperm and tissue extraction, all prawns were anesthetized in ice, and sperm collection and their processes were followed the method described by Watthammawut et al. (2015) and Surinlert et al. (2016). Briefly, the testes and vas deferens were carefully dissected out from the prawn specimens and minced with a razor blade to obtain one mm-sized pieces. To release sperm, the tissues were agitated in phosphate-buffered saline (PBS) on an orbital shaker and then the suspension filtered through a 45-µm metal sieve (Endercotts, London, UK). The sperm samples were concentrated, repeatedly washed (500 ×g, 4 °C, 5 min), resuspended in PBS and placed on ice for use in subsequent experiments. For consistency in all following experiments, sperm were counted with a hemacytometer to obtain an experimental sample each with a concentration of  1 × 10^7^ cells/ml.

### Sequence comparison of CAT-D and phylogenic tree analysis

Since full-length of MrCAT-D was already deposited in the Genbank database (accession number KP262355.1), we thus conducted amino acid sequence comparison with the other 10 animal species using a Clustal Omega software (Thompson, Higgins & Gibson, 1994). The MrCAT-D amino acid sequence was submitted to predict the signal sequence by SignalP 4.1 server (http://www.cbs.dtu.dk/services/SignalP-4.1/) (Petersen et al., 2011) and *N*-linked glycosylation by NetNGlyc 1.0 server (Technical University of Denmark, Lyngby, Denmark). CAT-D amino acid sequences were obtained from NCBI through these following accession numbers: *Homo sapiens* (NP_001900.1), *Bos taurus* (NP_001159993.1), *Canis familiaris* (NP_001020792.1), *Xenopus laevis* (BAC57431.1), *Salmo salar* (ACH70630.1), *Pinctada maxima* (AEI58896.1), *Bombus impatiens* (XP_003489428.1), *Homarus americanus* (ACV53024.1), *Penaeus vannamei* (ROT71313.1), *Penaeus japonicus* (AIF27797.1), *Macrobrachium rosenbergii* (AMQ98967.1). The phylogenetic tree was performed using a Maximum likelihood method in a Molecular Evolutionary Genetic Analysis (MEGA) software version 7.0. The reliability was based on 1,000 replications where the percent bootstrap confidence value was indicated at each branch node (Kumar, Stecher & Tamura, 2016).

### Reverse transcriptase polymerase chain reaction (RT-PCR)

Primers used for both RT-PCR and in situ hybridization were designed from the existing MrCAT-D full sequence (Table 1 and Fig. S1). Various tissues used for RNA extraction including testis (Tes), vas deferens (Vas), terminal ampoule (Ter), Muscle (Mus), hepatopancreas (Hep), stomach (Sto), heart (Hea), gill (Gil), testicular sperm (Tsp), vas deferens sperm (Vsp) and spermatophore (Ssp) were placed in the tubes and immediately homogenized in Trizol reagent (Invitrogen, Carlsbad, CA) for RNA extraction following the manufacturer’s protocol (Direct Zol-RNA miniprep, Zymoresearch, CA). Briefly, tissue homogenate was centrifuged and the supernatant was transferred into the RNase-free tube. Equal volume of ethanol (95–100%) was added and gently mixed and transferred to the column. The DNA digestion buffer which contained DNase-I was added and incubated (30 min). The obtaining RNA was washed with washing buffer and loaded into the column. The RNA was eluted from the column by DNase/RNase-free water and its concentration was measured by Nano drop-2000C spectrophotometer (Thermo Scientific, Massachusetts). Thereafter, 2 µg of total RNA was converted to first-strand cDNA by a RevertAid First Strand cDNA synthesis kit (ThermoFisher Scientific Inc. Massachusetts) following the manufacturer’s protocol using oligo-dT-adaptor primer and Multi Serobe™ reverse transcriptase (200 U/ µL). The mixtures were amplified at 25 °C for 5 min, 42 °C for 60 min, 70 °C for 5 min and 4 °C, respectively. To amplify target DNA sequence, the first-stranded cDNA was synthesized from mRNA isolated from prawn tissues and used as the template for PCR amplification. The oligonucleotide primers (Pacific Science, Bangkok, Thailand) used in this study were shown in Table 1. *β*-actin of *M. rosenbergii* was used as reference gene (Priyadarshi et al., 2015). These primer sequences were designed and selected by using NCBI/primer blast and Clustal Omega/multiple sequence alignment. The PCR reaction was initiated with a cycle of denaturation at 94 °C (5 min), followed by 35 cycles of denaturation at 94 °C (30 s), annealing at 57 °C (30 s) and extension at 72 °C (30 s) and further extension at 72 °C (10 min). The PCR products (predicted product length = 323 bp) were checked by 1.2% agarose electrophoresis and stained with ethidium bromide followed by visualization under a GelDoc 1,000 (BioRad, Hercules, CA). The relative density of these PCR bands from the triplicated experiments were calculated by ImageJ software and plotted as mean band density. Occasionally, these PCR products were further verified their sequences by Macrogen (Seoul, Republic of Korea).

**Table 1:** Oligonucleotide primers used for RT-PCR amplification and probe synthesis for in situ hybridization.

**Primer**	**Nucleotide sequence**	**Purpose**
CatD-F	5′-CCTGTTTTCTACAATATGGTTA-3′	RT-PCR expression analysis and In situ hybridization
CatD-R	5′-GGCTTAGCACCAATCTTCTTGTTG-3′	
*β*-actin-F	5′-ATTGGACTTCGAGCAGGAGA-3′	Reference genes
*β*-actin-R	5′-ACAGGTCCTTACGGATGTCG-3′	

### Immunohistochemistry with anti-CAT-D and phalloidin

The paraffin sections of testes collected from mature blue claw male giant freshwater prawns (aged >6 months, weighed 100-150 gm) were deparaffinized with series of xylene and rehydrated in a decreasing grade ethanol (100% to 70%). The sections were treated with 1% hydrogen peroxide (H_2_O_2_) to quench endogenous peroxidase and with 4% BSA in 0.1 M glycine in PBS to block free aldehyde and non-specific antibody binding. The tissues were further treated 0.1 M citrate buffer, pH 6.0 at 60 °C and 0.5% Triton-X 100 in PBS for antigen retrieval and tissue permeabilization, respectively. The sections were exposed to rabbit anti-human CAT-D polyclonal antibody (EMD Merck, Darmstadt, Germany) at a dilution of 1:200 (4 °C, overnight) followed by exposing the sections to horseradish peroxidase (HRP) conjugated goat anti-rabbit IgG at dilution of 1:400 (4 °C, 1 hr). It should be noted that the sequence similarity between heavy chain of human CAT-D (used as antigen to generate antibody) and MrCAT-D is about 58% (Suppl Fig. 2). Enzymatic reaction was developed using 3, 3′- diaminobenzidine (DAB) and the reaction was stopped by an excessive rinsing in the distilled water. The sections were mounted and visualized under an Olympus BX53 microscope.

To staining the filamentous actin, 5-µm thick-paraffin sections of *M. rosenbergii* testes were processed under similar conditions mentioned above. The sections were permeabilized (room temperature, 10 min) with 0.5% TritonX-100 in PBS and blocked non-specific staining with 4% BSA. They were then incubated with Alexa 594 conjugated phalloidin (ThermoFisher Scientific, Waltham, MA) in 1% BSA at the dilution of 1:200 (30 min, room temperature). Fluorescent signals were acquired by an Olympus FV10i confocal microscope.

### Immunofluorescent staining of isolated sperm

For loose sperm staining, the sperm were processed either live or fixed with 2% paraformaldehyde (30 min, room temperature). After washing in PBS, the cells were treated with 2% BSA to block non-specific antibody binding and incubated (4 °C, 1 hr) with 1:200 rabbit anti-CAT-D polyclonal antibody and centrifuged (500 × g, 5 min) to wash away the unbound antibody. The sperm were further incubated (4 °C, 30 min) with 1:400 goat anti-rabbit IgG conjugated with Alexa-488 (Abcam, Cambridge, UK). The cells were washed twice with PBS (500 × g, 5 min), mounted with 50% glycerol (v/v) and visualized by an Olympus FV10i confocal microscope.

### In situ hybridization

The specific-DNA probe for *Macrobrachium rosenbergii* CAT-D was constructed by PCR-DIG labelling kit (Promega, Madison, WI). The reaction mixture contained 1 × PCR buffer, 1 × dNTP mixture (DIG-dUTP), Taq DNA polymerase and CAT-D forward and reverse primers (Table 1). The probe concentration was measured by Nanodrop-200C spectrophotometer and analyzed with 1.5% agarose gel. Testis sections were dewaxed, rehydrated in graded series ethanol (100%–70%) and incubated with TNE buffer (50 mM Tris–HCl, 10 mM NaCl, 1 mM EDTA, pH 7.4) containing 10 µg/ml RNase-free proteinase K (37 °C, 15 min). After treating with DEPC-PBS containing 4% paraformaldehyde, the sections were incubated (37 °C, 2 hr) with prehybridization buffer (4 × SSC: 600 mM NaCl, 60 mM Na-Citrate in DEPC-H_2_O, 50% deionized formamide). They were then treated with hybridization buffer (50% deionized formamide, 50% dextran sulfate, 50 ×Denhardt’s solution, 20 × SSC, 10 mg/ml denatured salmon sperm DNA) containing 100 ng of DIG-labeled DNA probe (42 °C, overnight, humid chamber). The probes for *MrCAT-D* were designed from primers CatD-F: 5′-CCT GTT TTC TAC AAT ATG GTT A -3′ and CatD-R: 5′- GGC TTA GCA CCA ATC TTC TTG TTG-3′ (Table 1) with the expected probe length of about 320 nucleotides. Negative control sections were incubated without DIG labelled MrCAT-D probe and processed under the same conditions as described above. The slides were washed and blocked a non-specific staining with 10% normal goat serum in PBS and incubated (2 hr, room temperature) with the anti-DIG antibody conjugated with alkaline phosphatase (1:500) in a humid chamber. Enzymatic products were developed with NBT/BCIP substrate (Promega, Madison, WI) and the reaction was stopped with TE buffer (10 mM Tris–HCl, 1 mM EDTA, pH 8.1). The sections were mounted with 50% glycerol in PBS (v/v), observed and photographed under an Olympus BX53 microscope.

### Fluorogenic enzyme assay of MrCAT-D and its hydrolysis of filamentous actin

We tested the function of MrCAT-D in hydrolyzing the specific fluorogenic CAT-D substrate and filamentous actin extracted from SH-SY5Y neuronal stem cells (a kind gift from Assoc. Prof. Permphan Dharmasaroja, NeuroScience laboratory, Department of Anatomy, Faculty of Science, Mahidol University). MrCAT-D enzymatic activity was measured from testicular extracts (TE) and Tsp lysates using a fluorogenic enzyme assays. CAT-D specific substrate was fluorogenic-4-methylcoumarin-7-amide (MCA)-Gly-Lys-Pro-Ile-Leu-Phe-Phe-Arg-Leu-Lys (Dnp)-D-Arg-NH_2_) (Enzo Life Sciences, Lausen, Switzerland). The reaction mixtures consisted of 10 µl (10 µg) of protein samples, 80 µl cathepsin assay buffer (0.1 M NaoAc, 0.2 NaCl, pH 3.5) and 10 µl of 20 µM fluorogenic substrates. The activities were monitored at 5-60 min at the excitation and emission wavelengths of 340 nm and 420 nm, using a fluorescent microplate reader (Tecan Spark, Männedorf, Switzerland). The enzyme activity was calculated based on AMC standard curve. One unit of enzyme activity was defined as micromoles of AMC released per min at 25 °C. The specific activity of enzyme was defined as units per mg protein. Inhibition assay of the enzyme was performed using 1 mM of CAT-D inhibitor, pepstatin A (a competitive type inhibitor that is selective for aspartic proteases; EMD Merck, Darmstadt, Germany). The inhibitor was pre-incubated with the protein samples for 30 min at room temperature before performing the enzyme assay. The remaining enzyme activities were measured in the same conditions as mentioned above.

To determine the function of MrCAT-D in hydrolyzing filamentous actin, 10 µg of SH-SY5Y proteins were incubated with 1) 100 µg of Tsp lysates, 2) 100 µg of TE extracts, 3) 0.2 µg of purified human liver CAT- D and 4) samples 1 and 2 pre-incubated 30 min with 1mM pepstatin-A inhibitor prior to the reaction. The mixture of reaction was allowed to stand at 37 °C (24 hr) in an incubation buffer (50 mM sodium acetate buffer, pH 5.5 containing 12.5 mM NaCl, 1.5 mM dithiothreitol and 7.7 mM sodium azide). The treated samples were subjected to 12.5% SDS-PAGE and either stained with Coomassie blue staining (for protein profiling) or Western blotting. Briefly, the membrane was treated with 5% skimmed milk and probed (2 hr, room temperature) with 1 µg/ml anti-actin or anti-GAPDH monoclonal antibody (Cell Signaling Inc., Beavery, MA) followed by HRP-conjugated corresponding secondary antibody (1:2,000 dilution, 1 hr, room temperature). The antigen-antibody complex was visualized by an enhanced chemiluminescent kit (Amersham Pharmacia, Buckinghamshire, UK). Analysis of the band densities in all samples was performed in the same pixel areas (490 ×765 pixels) using an ImageJ software (http://www.imagej.nih.gov/ij).

## Results

### Sequence analysis of MrCAT-D and its phylogenetic analysis

**Figure 1:**
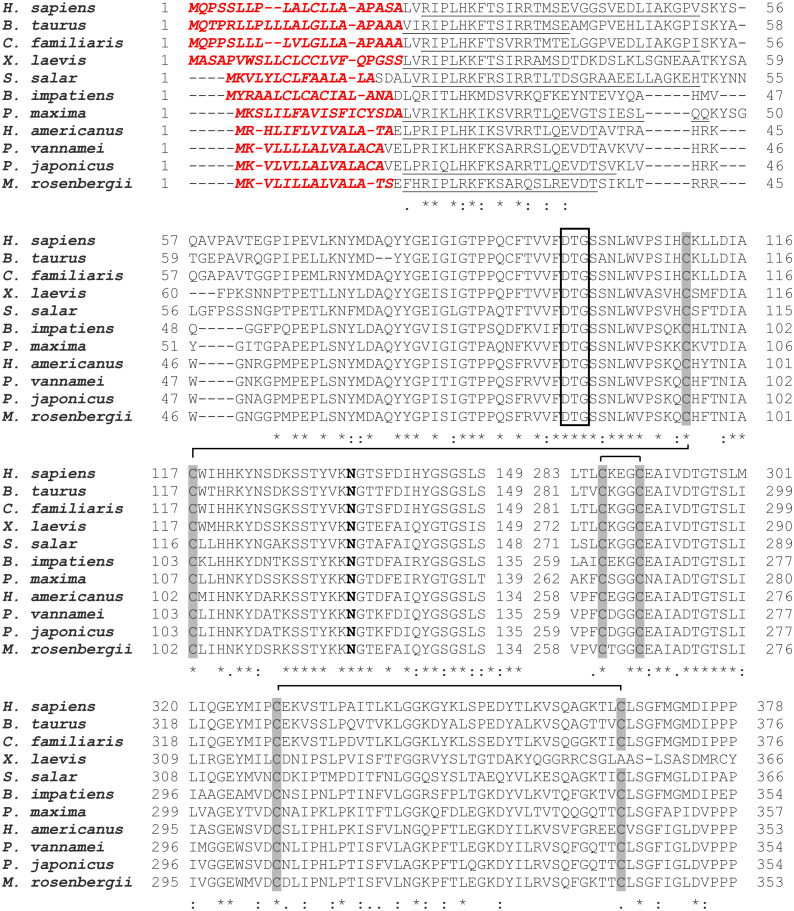
Alignment of amino acid sequences of MrCAT-D in M. rosenbergii with 10 other species. Accession numbers in databank are as follows: *H. sapiens* (NP001900.1), *B. taurus* (NP001159993.1), *C. familiaris* (NP001020792.1), *X. laevis* (BAC57431.1), *S. salar* (ACH70630.1), *B. impatiens* (XP003489428.1), *P. maxima* (AEI58896.1), *H. americanus* (ACV53024.1), *P. vannamei* (ROT71313.1), *P. japonicus* (AIF27797.1), *M. rosenbergii* (AMQ98967.1). The symbols in the comparison chart include signal peptide residues (italic-bold red letters); propetide cleavage sites (underlined); 6 cysteine residues (gray shading) that are linked by disulfide bridges; *N*-glycosylation site (NXS/T consensus sequence) at Asn119 (bold); and catalytic motifs (boxed). (*), (:), (.) indicate identical, conserved and semi-conserved amino acid residues.

As mentioned above that full-length of MrCAT-D sequence was already available in Genbank database (accession number KP262355.1), therefore, only sequence comparison with 10 different species, from crustaceans to mammals, was reported herein (Fig. 1). Generally, MrCAT-D consisted of 1,361 nucleotides which was translated into 385 deduced amino acid residues. The first 15 amino acids (1–15) at *N*-terminal sequence represented putative signal peptide (italicized, bold, red letters) and there was a cleavage site of putative pro-peptide (17–37, underlined) that made up of a single chain of pro-CAT-D protein, known to be an intermediate form of CAT-D. Following proteolytic processing, two cleavable chains of MrCAT-D was expected including an expected light chain with the amino acids between Trp46-Tyr116 (71 amino acids, calculated mass of 8.9 kDa) and expected heavy chain between Asp118-Ala385 (268 amino acids, calculated mass of 28.4 kDa). The putative catalytic motif was localized at Asp82, Thr83 and Gly84 (empty boxed amino acids). MrCAT-D had six cysteine residues to form three putative disulfide bonds which were Cys94-Cys102, Cys261-Cys265 and Cys304-Cys341 (gray box). Finally, the enzyme had one *N*-glycosylation site which was predicted at Asn119 (bold).

**Figure 2:**
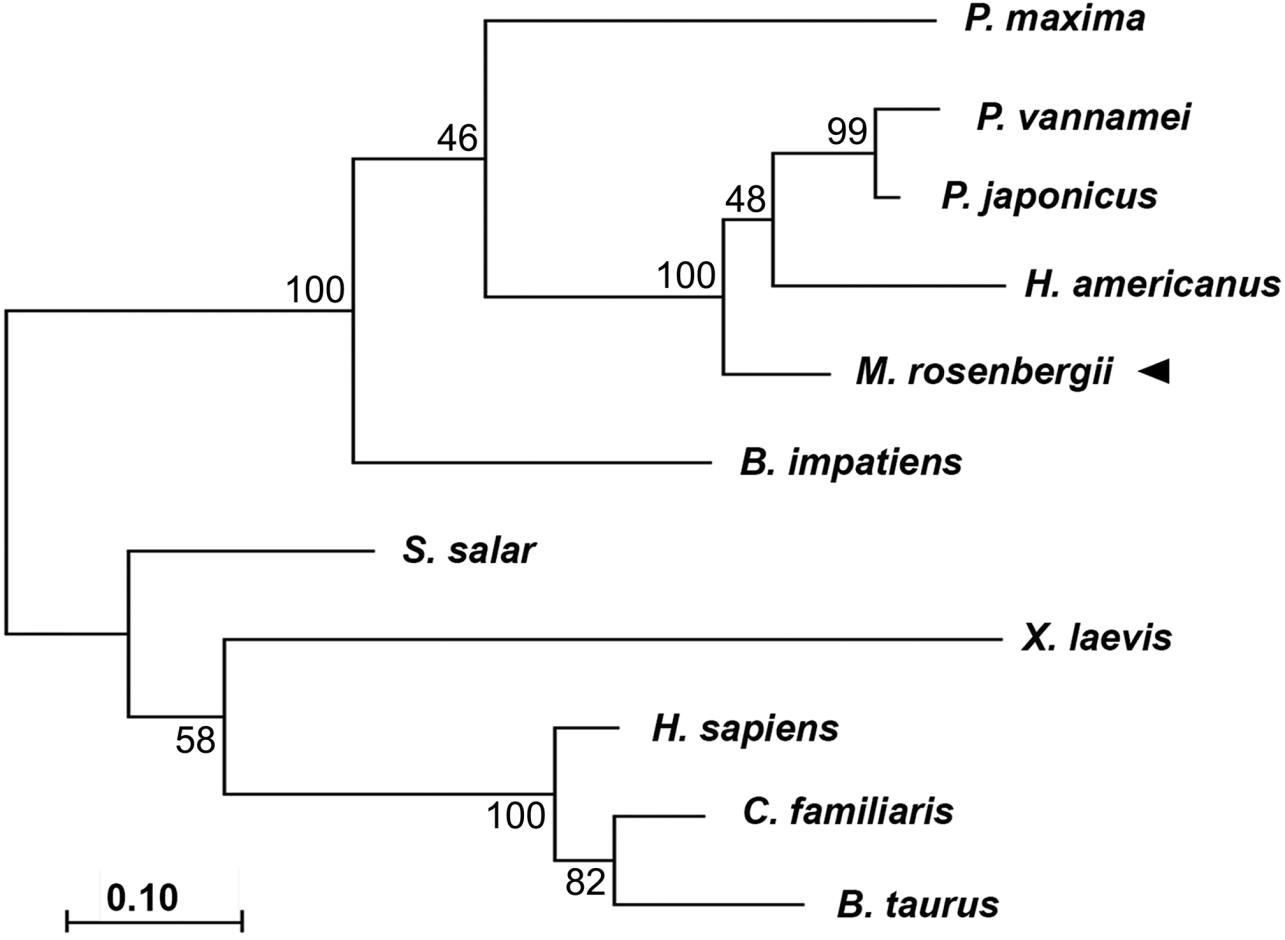
Claudogram of MrCAT-D sequences in comparison with those the other species, from crustaceans towards mammals. The trees were produced by a Maximum-likelihood method using the MEGA software version 7.0 and were based on the multiple sequence alignment shown in Fig. 1. Percent bootstrap values are indicated on the branches.

Phylogenic tree analysis based on a Maximum Likelihood method was used to compared CAT-D amino acid sequences from different species (Fig. 2). MrCAT-D showed high amino acid similarity to *P. japonicus* and *H. americanus* with 88% and 87% identities, respectively. The neighbor-joining tree of CAT-D sequences showed the four distinct subgroups: (1) vertebrate CAT-D, (2) insecta CAT-D, (3) mollusca CAT-D and (4) crustacean CAT-D. The result clearly revealed high conservation of CAT-D within three prawn/shrimp species, namely, they clustered within a single clade of the phylogenetic tree. It should be noted that CAT-D shared its ancestral origin towards many mammals including humans (*H. sapiens*) where the similarity of 57.58% was noted.

### Distribution of MrCAT-D gene in prawn tissues

**Figure 3:**
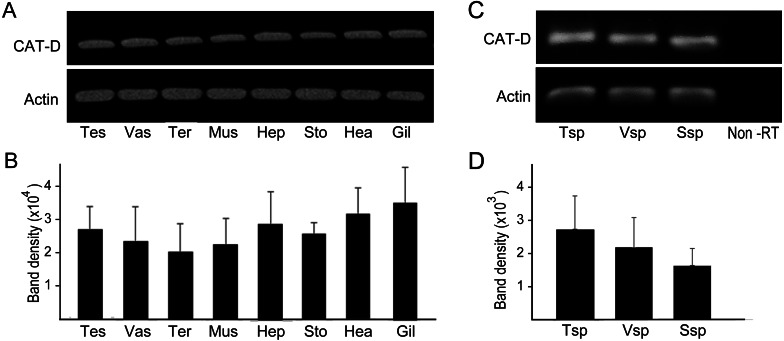
PCR amplification of MrCAT-D gene in multiple *M. rosenbergii* tissues and sperm from different part of reproductive tract. PCR amplification of MrCAT-D gene in multiple *M. rosenbergii* tissues (A) and sperm collected from different parts of reproductive tract (C). The deduced 323 bp of a single MrCAT-D band was amplified by primers CatD-F and CatD-R (see Table 1 for primers’ sequences) designed from the existing *MrCAT-D* sequence available in database as aforementioned. *Actin* gene was used as internal control. Densitometric analysis of the 323 bp bands in all tissues (B) and sperm cells (D) was calculated from triplicated experiments and expressed as mean ± S.D. Tes, testis; Vas, vas deferens; Ter, terminal ampule; Mus, muscle; Hep, hepatopancreas; Sto, stomach; Hea, heart; Gil, gill; Tsp, testicular sperm; Vsp, vas deferens sperm; Ssp, spermatophoric sperm; Non-RT, non-reverse transcriptase.

RT-PCR amplification was performed to show distribution of MrCAT-D in various sources of tissues, including reproductive related tissues - testis, vas deferens, terminal ampule, and other somatic tissues - muscle, hepatopancreas, stomach, heart and gill. The results in Figs. 3A and 3B revealed an intense, single PCR band at the expected 323 bp in all tissues studied, particularly those of reproductive related tissues (lanes 1–3) with the band density of about 2.0–2. 7 × 10^4^ arbitrary units. Of particular interest, we further confirmed that MrCAT-D transcripts were also present in sperm cells collected from testis (Tsp), vas deferens (Vsp) and spermatophore sperm (Ssp) (Figs. 3C, 3D) with the band density of about 1.7–2. 7 × 10^3^ arbitrary units suggesting self-biogenesis of MrCAT-D transcripts by developing germ cells in the testis which was rather different from mouse and human cases reported earlier (Saewu et al., 2012; Asuvapongpatana et al., 2013). The single 212-bp *β*-actin band, as internal controls, was evenly distributed in all tissues studied confirming an equal amount of RNA loaded in each tissue.

### Localization of MrCAT-D transcript and protein in prawn testis

**Figure 4:**
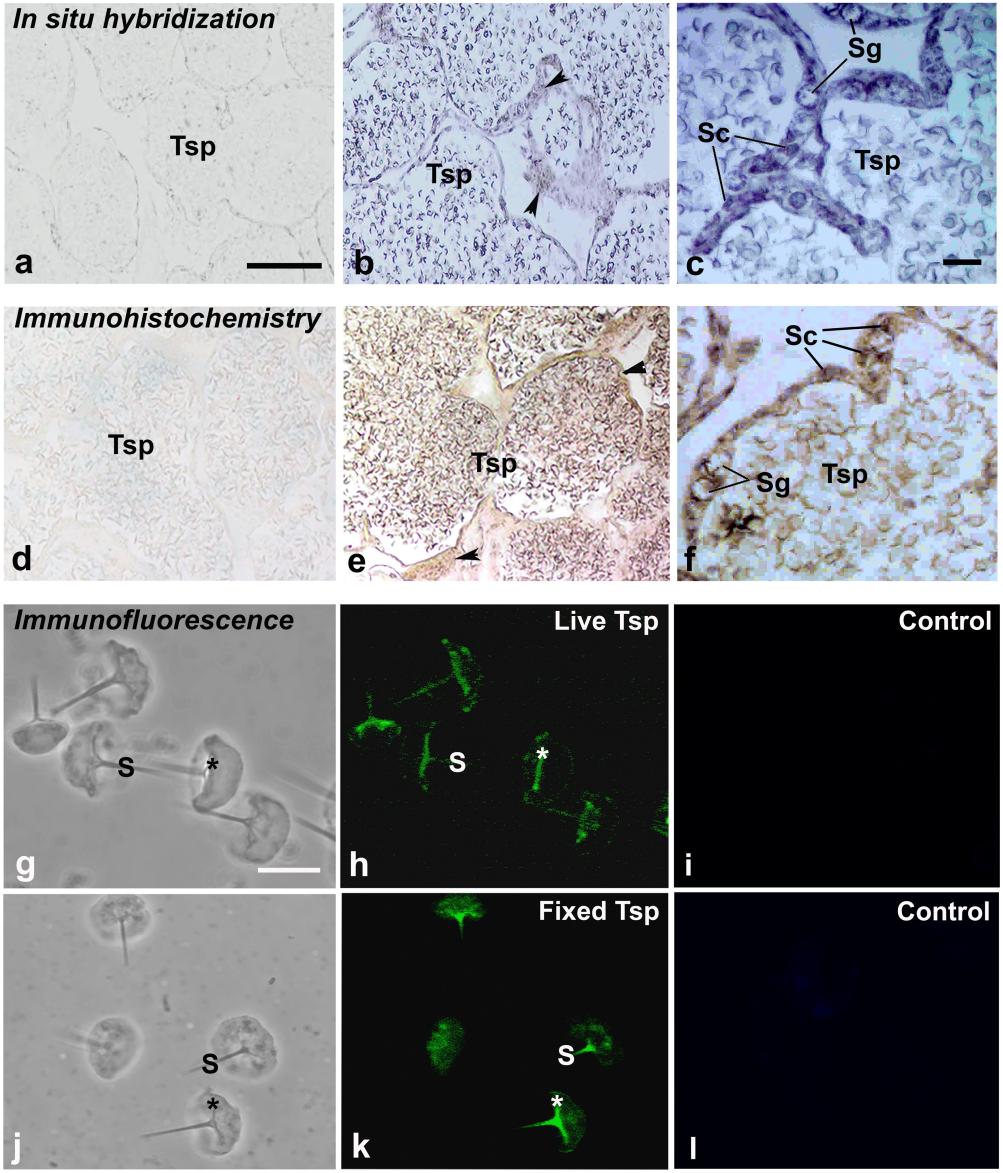
Multimodal approaches for visualizing localization of MrCAT-D transcript or protein in *M. rosenbergii* tissues or isolated testicular sperm. *MrCAT-D* mRNA was localized by in situ hybridization (B, C) while its protein was visualized by immunohistochemistry (E, F) or indirect immunofluorescence (H, K). Note the intense staining of anti-CAT-D in testicular sperm (Tsp), spermatogonia (Sg) and supporting epithelial cells (Sc and arrowheads) as well as in the isolated live (G–I or fixed sperm (J–L)) at their anterior spikes (S) and crescent bases (*). Bars in, A–B and D–E = 100 µm, C and F = 25 µm; A–L in g–l = 10 µm.

Using in situ hybridization, the transcripts of *MrCAT-D* were detected using the specific DIG-labelling oligonucleotide probes designed from *MrCAT-D* sequence. Specifically, an intense staining of MrCAT-D probe was detected within the supporting cells (Sc, elongated nuclei) and spermatogonia (Sg, round nuclei) (Ruiz et al., 2020) that lied against basal laminar of testicular tubules (Figs. 4B and 4C). Developing germ cells in the adluminal compartment were also intensely labelled with MrCAT-D probe, suggesting that both the somatic epithelial cells and germ cells actively synthesized MrCAT-D mRNA. Negative control of testicular section showed only a background staining (Fig. 4A).

To confirm the expression of MrCAT-D at the translational level, we used anti-CAT-D polyclonal antibody to visualize CAT-D protein expression in the prawn tissues. The markedly intense anti-CAT-D reactivity (as a dark brown enzymatic product) was found in the supporting cells (Sc) lining testicular tubules, clump of spermatogonia (Sg) and in Tsp cells with different degrees of staining (Figs. 4E and 4F). To further verify CAT-D localization in the sperm cells, we carried out indirect immunofluorescence (IIF) of live sperm (whose membranes remain intact and impermeable to antibody) and aldehyde fixed sperm (highly permeable to antibody). The IIF results (Figs. 4H and 4K) clearly demonstrated an intense staining of anti-CAT-D at the anterior spike (S) and at the crescent base (*) over the nuclei either in live or fixed sperm, confirming immunohistochemical results mentioned above (Figs. 4E and 4F). Negative controls of both live and fixed isolated cell staining exhibited minimal staining of antibody (Figs. 4I and 4L). The results thus suggest that CAT-D is a sperm inherent enzyme that is targeted to the membrane upon its biosynthesis during germ cell development.

### Cathepsin activity in testicular extracts and sperm and their hydrolysis over filamentous actin

**Figure 5:**
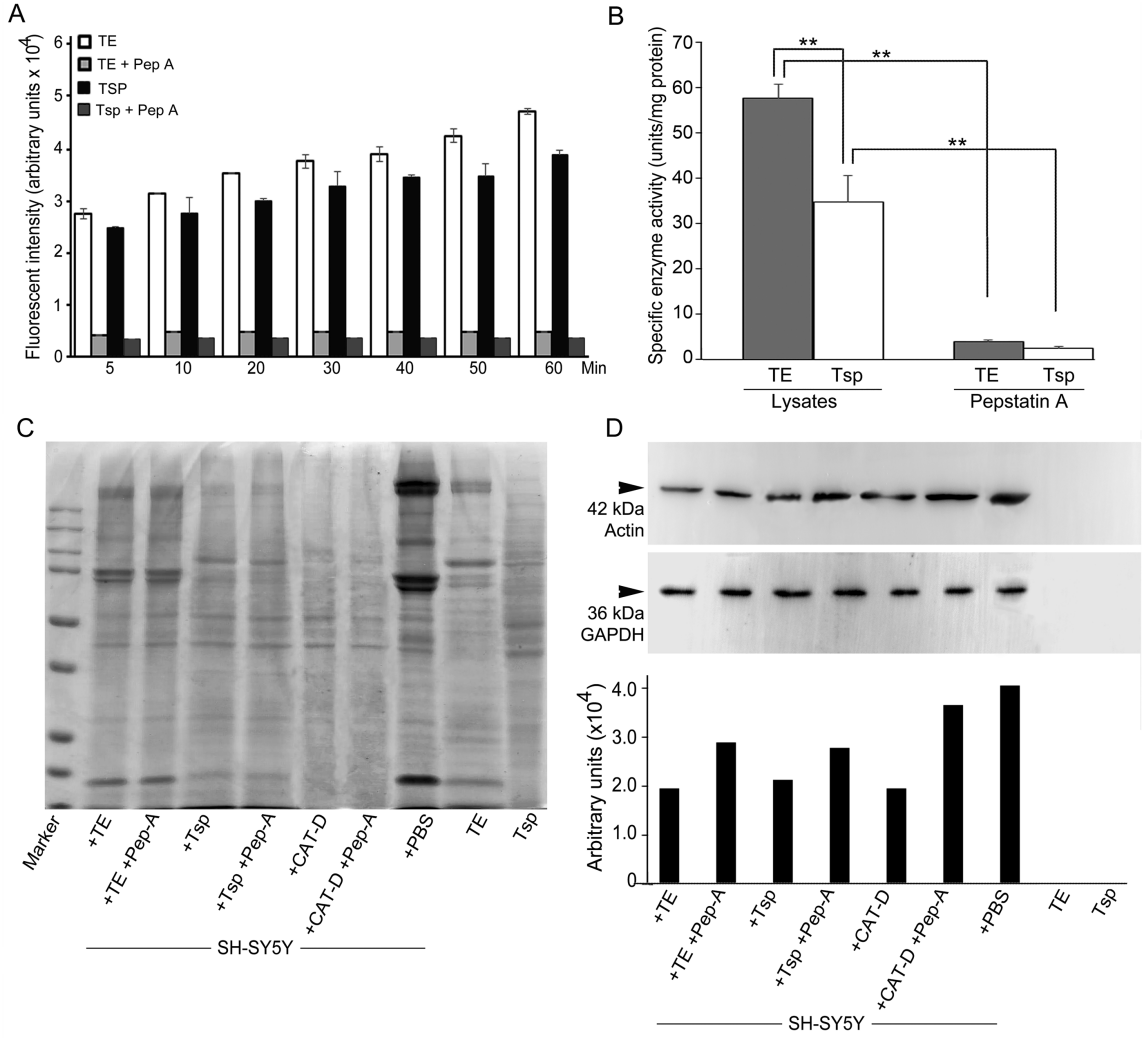
Activity of MrCAT-D in tissue extracts and their hydrolysis over the filamentous actin isolated from the cultured neuronal cells. The enzyme activity (A) of whole testis (TE) or testicular sperm (Tsp) over the CAT-D-specific fluorescent substrate (with or without pepstatin-A inhibitor) was measured at 5–60 min after addition of tissue extracts. The specific activity (B) was calculated at 60 min using the substrate concentration of 20 µM and expressed as units per mg protein. The ability of MrCAT-D to digest filamentous actin from neuronal, SH-SY5Y, cells were demonstrated either by Coomassie blue staining (C) or Western blotting (D) using monoclonal anti-actin or anti-GAPDH antibody. Densitometric analysis of all samples is shown in the lower panel. Lanes 1–7 represent SH-SY5Y extracts exposed to TE (lane 1); TE + pepstatin-A (lane 2); Tsp (lane 3); Tsp + pepstatin-A (lane 4); purified CAT-D (lane 5); CAT-D + pepstatin-A (lane 6); non-treated or PBS (lane 7). TE and Tsp extracts are shown in lanes 8–9.

We further checked MrCAT-D enzyme activity in Tsp and TE extracts using the specific fluorogenic CAT-D substrate (MCA-Gly-Lys-Pro-Ile-Leu-Phe-Phe-Arg-Leu-Lys (Dnp)-D-Arg-NH_2_) with or without the presence of 1 mM pepstatin, a competitive aspartic enzyme inhibitor. The results revealed a considerable amount of the emitted AMC fluorescent intensity at many time points studied reflecting CAT-D activities in TE and Tsp extracts (Fig. 5A). The specific enzyme activity (determined when the substrate used was >5 fold of the *K*_m_ of CAT-D (Yasuda et al., 1999)) was about 57.8 ± 2.2 and 34.9 ± 5.8 units/mg protein, respectively (Fig. 5B). These activities could be drastically abolished (<4.00 units/mg protein) when pepstatin was present in the incubates, verifying the existence of MrCAT-D in the epithelial cells of testicular tubules as well as sperm cells.

To further tested the function of MrCAT-D, we exploited protein extracts from SH-SY5Y neuronal cells (due to their enrichment in cytoskeletal proteins including actin filaments) and further exposed them to either TE or Tsp extracts or purified human CAT-D (positive control). As shown in Fig. 5C, there was a minimal change in the protein patterns of all sample studied, even when the protein extracts were exposed to purified CAT-D enzyme (engaging a rather strong hydrolytic activity). Therefore, digestion of filamentous actin was further checked by the reactivity of anti-actin antibody with the filamentous actin left after MrCAT-D hydrolysis (Fig. 5D). It should be first noted that monoclonal anti-actin that we used was rather specific to mammalian (SH-SY5Y) actin while it showed no reactivity with shrimp actin that we tested at all (Fig. 5D, lanes TE and Tsp). The intensity of an immunoreactive band at 42 kDa (a molecular mass of filamentous actin) was markedly reduced upon hydrolyzed by TE or Tsp extracts in the similar trend as that of purified human CAT-D, although not as much as the latter group. Upon pre-treating Tsp, TE extracts and purified CAT-D with pepstatin A, a higher intensity of the 42-kDa anti-actin reactive bands was seen (lanes +Pep-A), suggesting a less digestion of filamentous actin by MrCAT-D in the tissues under the influence of its inhibitor. Densitometric analysis further confirmed reduction of anti-actin reactive band intensities in SH-SY5Y samples treated with TE, Tsp and purified CAT-D compared with either those inhibited by pepstatin-A or non-treated SH-SY5Y protein. The densities ranged from 1.8-2. 0 ×10^4^ arbitrary units in those treated samples where they were 2.6-3. 5 ×10^4^ arbitrary units in pepstatin-A inhibited samples and 3. 9 ×10^4^ arbitrary units in non-treated SH-SY5Y sample (Fig. 5D, lower panel). It should also be noted that the intensity of the 42-kDa band inhibition by pepstatin-A (Fig. 5D, lanes 2, 4, 6) was relatively lower than the starting concentration of the actin band (lane 7), thus a complete inhibition could not be claimed.

### Presence of actin filamentous meshwork in between sperm and supporting cells

**Figure 6:**
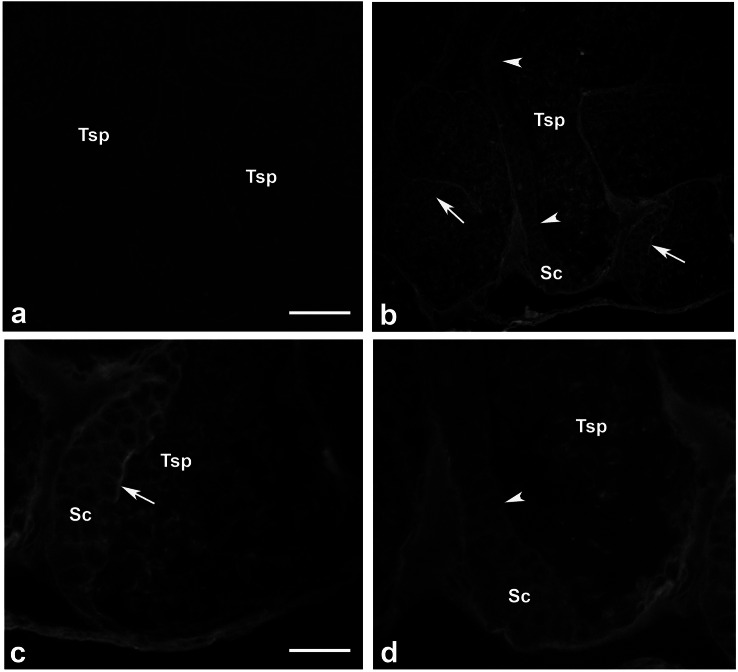
Existence of filamentous actin patches in between testicular sperm and supporting epithelial cells. Testes of M. rosenbergii were probed with phalloidin conjugated to Alexa 594 and the fluorescent signals were acquired by confocal microscope (A–D). Note the intense staining patches of phalloidin at the apical surface of Sc and Sg in the places where Tsp still contact (C, arrow), while the staining of phalloidin is diminished in the area where Tsp are released into the lumen (D, arrowhead). Bar in A = 100 µm, Bar in C = 30 µm.

We investigated whether actin meshwork existed in the junction between apical aspect of supporting cells and testicular sperm, by probing with phalloidin (a well-known marker for filamentous actin). The results in Fig. 6 showed an intense phalloidin staining as the patch-like structure in between spermatogonia, supporting cells and testicular sperm (Tsp; panel C, arrow), suggesting the presence of filamentous actin meshwork in this area. In addition, the intense staining patches of phalloidin disappeared at the sites where Tsp were detached from supporting epithelium and released into the lumen, leaving only a faint actin staining within the cells (panel D, arrowhead). This result thus provided a good clue on the presence of actin-rich meshwork at the junction between supporting and germ cells (similar to mammalian ES-like complex) (Cheng & Mruk, 2011; Pelletier, 2011). Interestingly, this result also suggested that actin meshwork must be disrupted or disappeared which should be in conjunction with the sperm release into the lumen of testicular tubules. As mentioned, presence of actin as the faint staining rings within epithelial cells and Tsp was also noted (Figs. 6C and 6D), which should represent basic cytoskeletal element that supports cellular architecture.

## Discussion

In mammals, existence and distribution of CAT-D in male reproductive tissues have been reported to be greatly variable, unlike a more straightforward pattern found in somatic cells where the enzyme is well-known to be a house-keeping enzyme in the lysosomes (Masson et al., 2010; Benes, Vetvicka & Fusek, 2008). Giving examples in mammalian epididymis, CAT-D is restrictedly localized to only principle cells of the tract epithelium while both clear and halo cells (which are fully loaded with lysosomes) are devoid of CAT-D expression (Saewu et al., 2012; Asuvapongpatana et al., 2013; Hermo & Andonian, 2003). A cell-specific localization has also been reported in testicular epithelium where only somatic Sertoli and Leydig cells are loaded with CAT-D, but not in the acrosome, a lysosome-equivalent structure, of mouse and human developing spermatids and testicular sperm (Saewu et al., 2012; Asuvapongpatana et al., 2013). However, this fact did not hold true in case of prawn, *M. rosenbergii*, reported herein. Our PCR and immunolocalization results (Figs. 3 and 4E, 4F) revealing the broad distribution of MrCAT-D in the supporting epithelial and spermatogenic cells in testis, expression the level of which was comparable to those found in other somatic tissues indicates uniqueness of MrCAT-D in prawn testes from those reported in mammals. The most striking difference was the presence MrCAT-D in spermatogonia and Tsp (apart from the supporting Sertoli-like cells) as their self-inherent transcriptional and translational products. Labelling of multi-cell types in testis by polyclonal antibody might raise the question of its non-specificity (Figs. 4E, 4F), however, the corresponding pattern of *MrCAT-D* transcripts localization (Figs. 4B, 4C) as well as RT-PCR amplification in Tsp (Fig. 3C) should provide firm evidence to support multi-cellular sources of MrCAT-D in prawn testis. In addition, we observed a similar anti-CAT-D staining on the live versus aldehyde-fixed sperm suggesting that CAT-D was favorably localized as a surface anchoring protein rather than the acrosomal resident protein (which otherwise should be additionally localized to the “ruffled” rim at the bottom end of the fixed sperm (Watthammawut et al., 2015). Two possible routes of enzyme trafficking to the sperm surface has been suggested –it could be either through its leakage via the physiological shunt from the acrosomal matrix (Monck & Fernandez, 1996; Foster et al., 1997) or via its post-translational trafficking as other membrane proteins upon its biogenesis. Although the latter regard is more favorable in our case since MrCAT-D is a self-inherent protein, however, a thorough investigation would be required before any firm conclusion can be made.

As MrCAT-D existed as surface membrane protein of Tsp, its physiological function in reproductive system could be interpreted in two aspects. In testicular environment, the essence of enzymes either from sperm surface enzymes or within supporting epithelial cells (likely lysosomal origin) would be significant for digesting the supporting elements or meshwork in between Tsp and their supporting cells as suggested in mammals (Yanagimachi, 1994; Cheng & Mruk, 2011). The most recognizable membrane specializations coupled between these two cells are the apical ectopic specialization (ES) and tubulobulbar complex (TBC), both of which are filamentous actin-rich apparatuses that support these membrane junctional complexes (Cheng & Mruk, 2011; Pelletier, 2011; Franca et al., 2012). Based on this structural feature, we were also evident here the presence of actin-enriched patches in between the junction of developing germ cells and the apical cytoplasm of supporting cells (Fig. 6), indicating the presence of actin-rich membrane specialization in prawn testicular epithelium. This claim was based on a high affinity of phalloidin probing which specifically recognizes filamentous actin (Dancker et al., 1975). Hydrolysis of this supporting actin framework by enzymatic activity would thus be required to free testicular sperm into tubular lumen within testes (Mruk & Cheng, 2004). In this aspect, one should presume that CAT-D would stand to be a good candidate to disrupt actin meshwork, due to its well-known hydrolyzing ability towards filamentous actin substrate (Hughes et al., 2000). In fact, our results on the enzymatic activity of sperm-derived MrCAT-D towards filamentous actin (Fig. 5D) would favor the hydrolytic function of this enzyme towards actin meshwork. Two aspects of enzymatic activity should be noted. Firstly, the fluorogenic substrate that we employed was rather specific to CAT-D (preferring hydrophobic amino acids (Phe-Phe) at cleavage site P1 and P1′ as well as polar amino acid (Arg) at P2′ and hydrophobic amino acid at P2 position (Pimenta et al., 2001). Secondly, the enzymatic activity that we showed herein may also be derived from other aspartic protease subclasses present in the extracts, due to cross-reactivity of pepstatin-A (used herein) among those aspartic group of enzymes (Gacko et al., 2007). In vas deferens environment (epididymis equivalent), surface MrCAT-D (if exists) would serve as a biomarker for sperm maturation or capacitation as part of post-testicular sperm modifications similar to that reported in mammals (Saewu et al., 2012; Asuvapongpatana et al., 2013). The surface existence of CAT-D in the distal tract would suggest plausibility of the enzyme to act as sperm ligand that interact with the egg receptors counterpart, a classical example that has been shown in sea urchins (Neill & Vacquier, 2004). This possible function of prawn MrCAT-D in vas deferens should still be kept concerned, although it is not a major emphasis in this study.

Together, we demonstrated MrCAT-D transcriptional and translational expressions in the supporting epithelial cells and spermatogenic cells in prawn testes. Inherent MrCAT-D is trafficked onto Tsp surface with a not-yet defined mechanism. Based on the function of MrCAT-D in hydrolyzing filamentous actin and the presence of filamentous actin meshwork in the vicinity between Tsp and their supporting cells, its involvement in disrupting filamentous actin meshwork to release Tsp into tubular lumen is proposed.

## Conclusions

MrCAT-D was detected transcriptionally and translationally in supporting epithelial cells and spermatogenic cells in prawn testis as well as on the sperm surface. Its function in hydrolyzing filamentous actin isolated from HY5Y neuronal cells was demonstrated. Since filamentous actin is known to be a major composition of ES complex, the hydrolytic function of MrCAT-D towards filamentous actin therefore suggest its possible involvement in releasing sperm from testicular epithelium, although its function in sperm maturation could not be excluded. Future research in knocking down MrCAT-D gene by siRNA are planned to conducted in our laboratory to verify its direct function in the prawn testis.

##  Supplemental Information

10.7717/peerj.10218/supp-1 Supplemental Information 1 Full sequence of MrCAT-D and amino acid positions of designed primers

10.7717/peerj.10218/supp-2 Supplemental Information 2 Alignment of amino acid sequences comparing between human CAT-D (heavy chain, as an antigen to generate polyclonal antibody) and MrCAT-D

10.7717/peerj.10218/supp-3 Supplemental Information 3 Raw data of the MrCAT-D band density in all tissues for Figure 3B

10.7717/peerj.10218/supp-4 Supplemental Information 4 Raw data of the MrCAT-D band density in sperm for Figure 3D

10.7717/peerj.10218/supp-5 Supplemental Information 5 Raw data of fluorescent intensity (AMC release) for Figure 5A

10.7717/peerj.10218/supp-6 Supplemental Information 6 Raw data of specific enzyme activity for Figure 5B

10.7717/peerj.10218/supp-7 Supplemental Information 7 The ability of MrCAT-D in tissue extracts to digest filamentous actin from neuronal, SH-SY5Y, cells as demonstrated by Coomassie blue staining

10.7717/peerj.10218/supp-8 Supplemental Information 8 The ability of MrCAT-D in tissue extracts to digest filamentous actin from neuronal, SH-SY5Y, cells as demonstrated by Western blotting

10.7717/peerj.10218/supp-9 Supplemental Information 9 Reactivity of monoclonal anti-GAPDH antibodyto SHSY5Y protein extract as an internal control

10.7717/peerj.10218/supp-10 Supplemental Information 10 Denstitometric analysis of anti-actin reactive bands relative to the density of anti-GAPDH reactive bands

## Additional Information and Declarations

### DNA Deposition

The following information was supplied regarding the deposition of DNA sequences: CAT-D data is available at GenBank: KP262355.1.

### Data Availability

The following information was supplied regarding data availability: Raw data are available in the Supplemental Files. Full sequence of MrCAT-D and the primer designed is supplied as Fig. S1. Alignment of the human CAT-D (heavy chain, used as antigen to generate antibody) with MrCAT-D is loaded as Fig. S2. Raw data about densitometric analysis in Figs. S3B, S3D and S5D and about enzymatic activity (Fig. 5A) and specific enzyme activity (Fig. 5B) are provided in Excel format. The ability of MrCAT-D to digest filamentous actin from neuronal, SH-SY5Y, is provided in Fig. 5C, or Western blotting using monoclonal anti-actin or anti-GAPDH antibody (upper and middle panels in Fig. 5D). Densitometric analysis of all samples is shown in the lower panel (Fig. 5D). The uncropped blots are available as Supplemental Files.
