# Supplementary material for: Cathepsin D in prawn reproductive system: its localization and function in actin degradation
Source: PeerJ. 2020 Nov 11;8:e10218. doi: 10.7717/peerj.10218 (PMC7666547; doi:10.7717/peerj.10218)
Supplement: Supplemental Information 1 [file peerj-08-10218-s001.pdf]

Supplementary Figure 1 Full sequence of MrCAT-D and primer design

>KP262355.1 Macrobrachium rosenbergii cathepsin-D (CAT-D) mRNA, complete cds
AGCCAACACCTGACGCTGACAGCTTCTCTACATGCACACTGCACAGTCGAGACTTCATTCTTCTTCTCGC
TATATCGTTGGTCTCTGTTTCTTTGTACTCTGGTGCTGAGGATGAAGGTGTTGATTTTACTGGCCCTGGT
GGCTCTTGCCACTTCGGAATTTCACAGAATTCCATTGCGAAAATTTAAATCTGCCAGGCAGAGTTTGAGA
GAAGTAGACACCTCCATAAAACTTACACGCCGCCGATGGGGAAATGGAGGTCCTATGCCCGAGCCTCTGT
CAAATTACATGGATGCACAGTACTATGGACCGATCAGCATTGGCACTCCTCCTCAGTCCTTTAGAGTTGT
ATTTGACACTGGCTCATCCAATCTCTGGGTTCCTTCCAAGCAGTGCCACTTTACCAATATTGCTTGCTTA
ATTCACAATAAGTACGATTCACGTAAGTCATCAACATACAAGAAGAATGGGACTGAATTTGCTATCCAGT
ATGGATCAGGGTCACTCTCAGGTTACTTGTCCACAGACACAGTTAGTGTTGGTTCAGTAGTTGTGAAAGA
CCAGACATTTGCAGAAGCTTTAAGTGAACCAGGAATGGCTTTTGTTGCAGCGAAGTTTGATGGTATTCTT
GGTATGGCCTATGACAGAATTGCAGTTGATGGAGTTACTCCTGTTTTCTACAATATGGTTAGCCAAAAGC
TTGTGCCAGCTCCAGTCTTCAGCTTCTATCTTAATAGAGATCCTAGTGCTCCTGAGGGTGGTGAGTTAAT
TCTTGGAGGTTCTGACCCAAAATACTATAAGGGAGACTTTACTTACCTACCAGTTGATCGTCAGGGCTAC
TGGCAGTTCAAAATGGACGGAGTGCAAATAGATGGAGCTGATGTTCCTGTCTGTACGGGTGGTTGTGAAG
CTATTGCTGATACTGGCACCTCACTCATTGCTGCCCCCTCAGAGGAAGCTCGTCTTATCAACAAGAAGAT
TGGTGCTAAGCCTATTGTTGGTGGAGAGTGGATGGTTGACTGTGATCTTATTCCCAATCTTCCTACTATT
TCATTTGTATTGAATGGGAAGCCATTTACCCTGGAAGGCAAAGACTACATTCTCAGGGTGAGTCAGTTTG
GAAAGACAACTTGCCTCTCTGGATTCATTGGACTTGATGTACCTCCACCCATGGGCCCCATGTGGATTCT
GGGTGATGTCTTCATTGGTCGATTCTACACTGAGTTTGACATGGAAAACAACAGGGTTGGCTTTGCCACT
GCTGCCTAGAAAGGAGCAAAGCAAAACCACTCTAGGTTTGTTGCTTTGTTCATCATTACTTCAGAAATTA
AAGTTTATCATCCCCAAAAAAAAAAAAAAAAA
