# Supplementary material for: Cathepsin D in prawn reproductive system: its localization and function in actin degradation
Source: PeerJ. 2020 Nov 11;8:e10218. doi: 10.7717/peerj.10218 (PMC7666547; doi:10.7717/peerj.10218)
Supplement: Supplemental Information 2 [file peerj-08-10218-s002.pdf]

<u>Supplementary Figure 2</u> Alignment of human heavy chain (NP_001900.1:169-412) and heavy chain of *Macrobrachium rosenbergii* CAT_D (AMQ98967.1:118-385) by Clustal Omega Program

---

```
H.sapiens               -------------------------LGGVKVERQVFGEATKQPGITFIAAKFDGILGMAY 35
M.rosenbergii           KNGTEFAIQYGSGSLSGYLSTDTVSVGSVVVKDQTFAEALSEPGMAFVAAKFDGILGMAY 60
                                                 :*.* *: *.*.** .:**::*:************

H.sapiens               PRISVNNVLPVFDNLMQQKLVDQNIFSFYLSRDPDAQPGGELMLGGTDSKYYKGSLSYLN 95
M.rosenbergii           DRIAVDGVTPVFYNMVSQKLVPAPVFSFYLNRDPSAPEGGELILGGSDPKYYKGDFTYLP 120
                         **:*:.* *** *::.****   :*****.***.*  ****:***:* *****.::**

H.sapiens               VTRKAYWQVHLDQVEVA-SGLTLCKEGCEAIVDTGTSLMVGPVDEVRELQKAIGAVPLIQ 154
M.rosenbergii           VDRQGYWQFKMDGVQIDGADVPVCTGGCEAIADTGTSLIAAPSEEARLINKKIGAKPIVG 180
                        * *:.***.::* *::  :.: :*. *****.******:..* :*.* ::* *** *::

H.sapiens               GEYMIPCEKVSTLPAITLKLGGKGYKLSPEDYTLKVSQAGKTLCLSGFMGMDIPPPSGPL 214
M.rosenbergii           GEWMVDCDLIPNLPTISFVLNGKPFTLEGKDYILRVSQFGKTTCLSGFIGLDVPPPMGPM 240
                        **:*: *: : .**:*:: *.** :.*. :** *:*** *** *****:*:*:*** **:

H.sapiens               WILGDVFIGRYYTVFDRDNNRVGFAEAARL  244
M.rosenbergii           WILGDVFIGRFYTEFDMENNRVGFATAA--  268
                        **********:** ** :******* **
```
