# Supplementary figures and images for: Cathepsin D in prawn reproductive system: its localization and function in actin degradation
Source: PeerJ. 2020 Nov 11;8:e10218. doi: 10.7717/peerj.10218 (PMC7666547; doi:10.7717/peerj.10218)

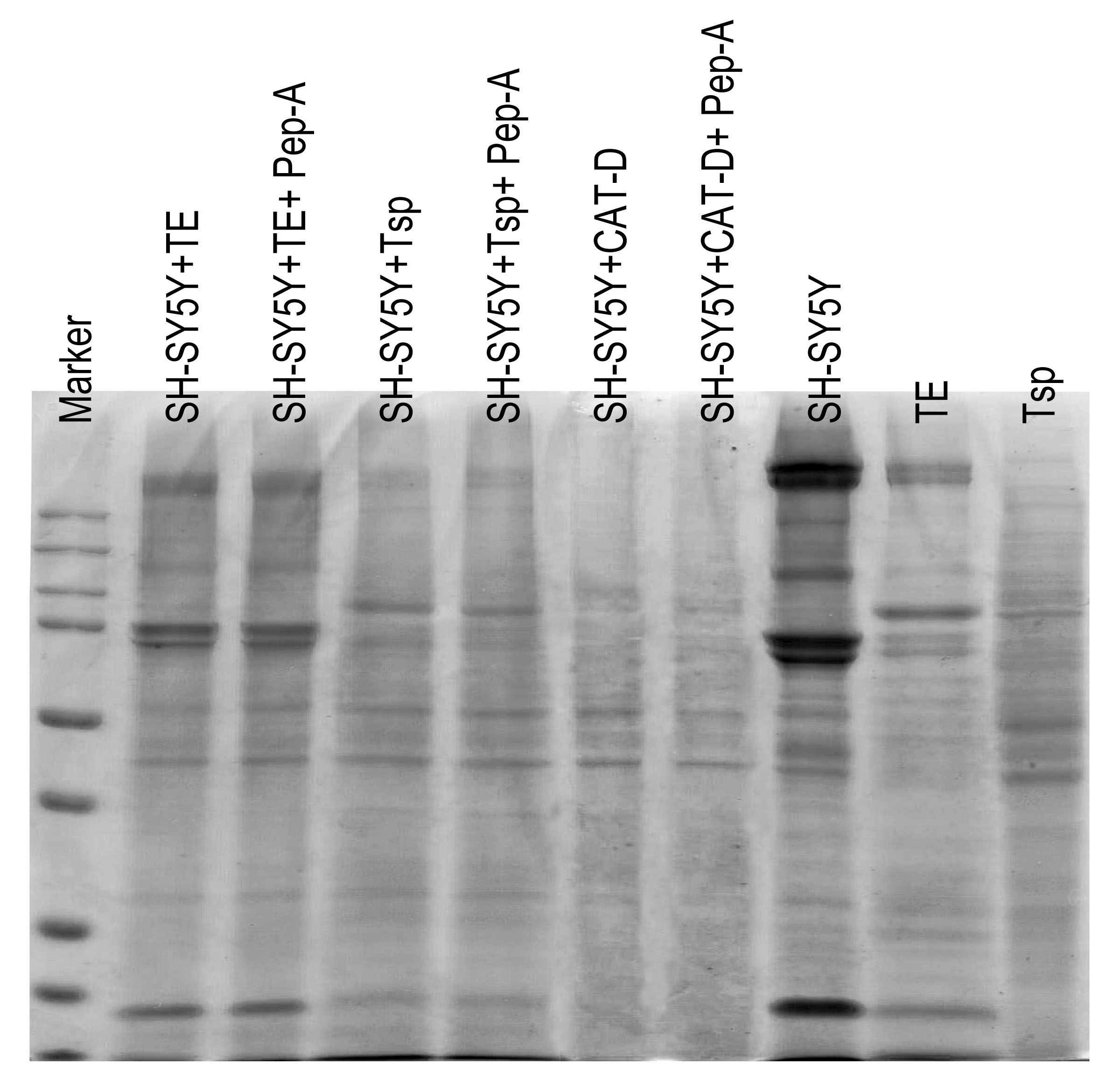

Supplement: Supplemental Information 7 [file peerj-08-10218-s007.jpg]

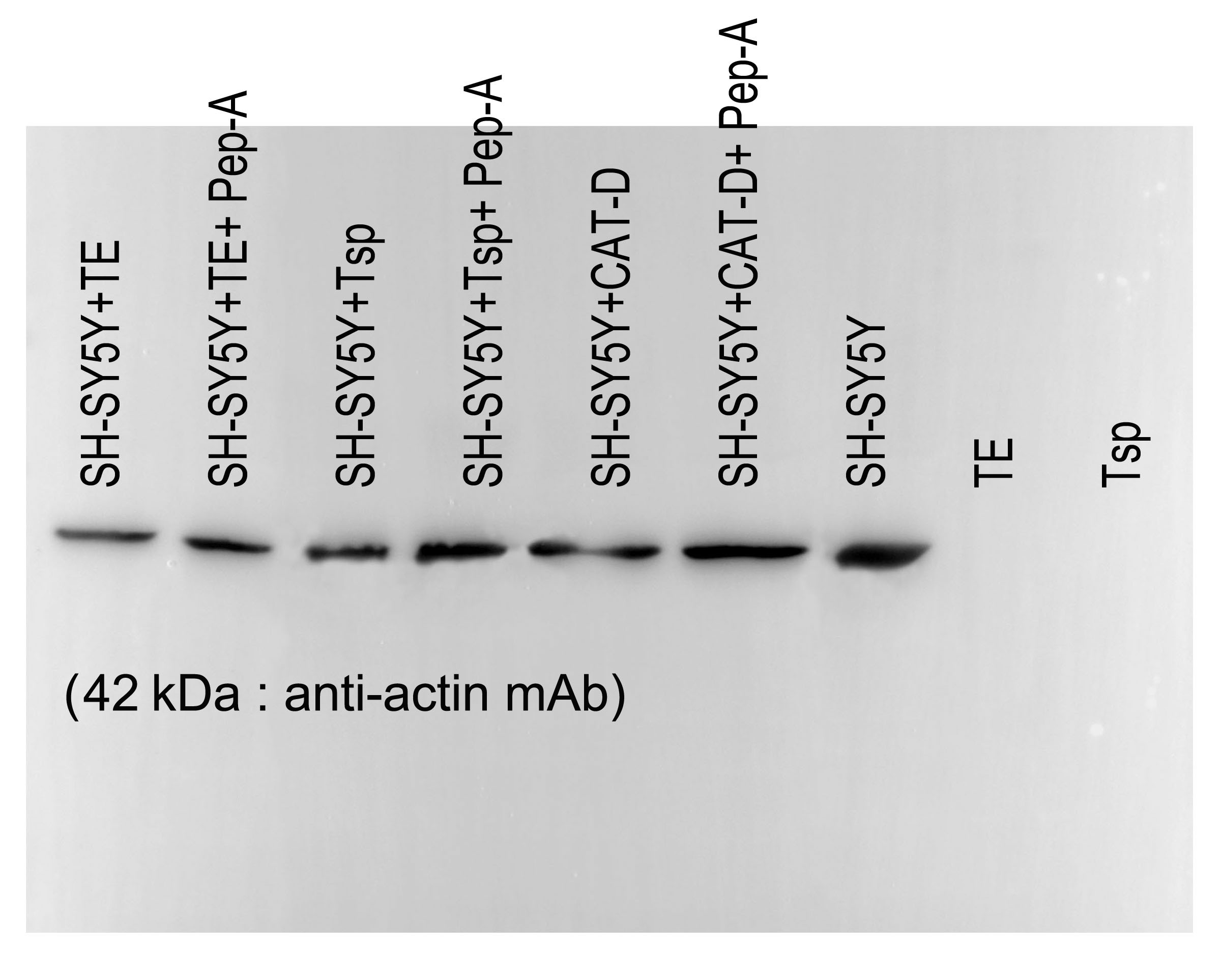

Supplement: Supplemental Information 8 [file peerj-08-10218-s008.jpg]

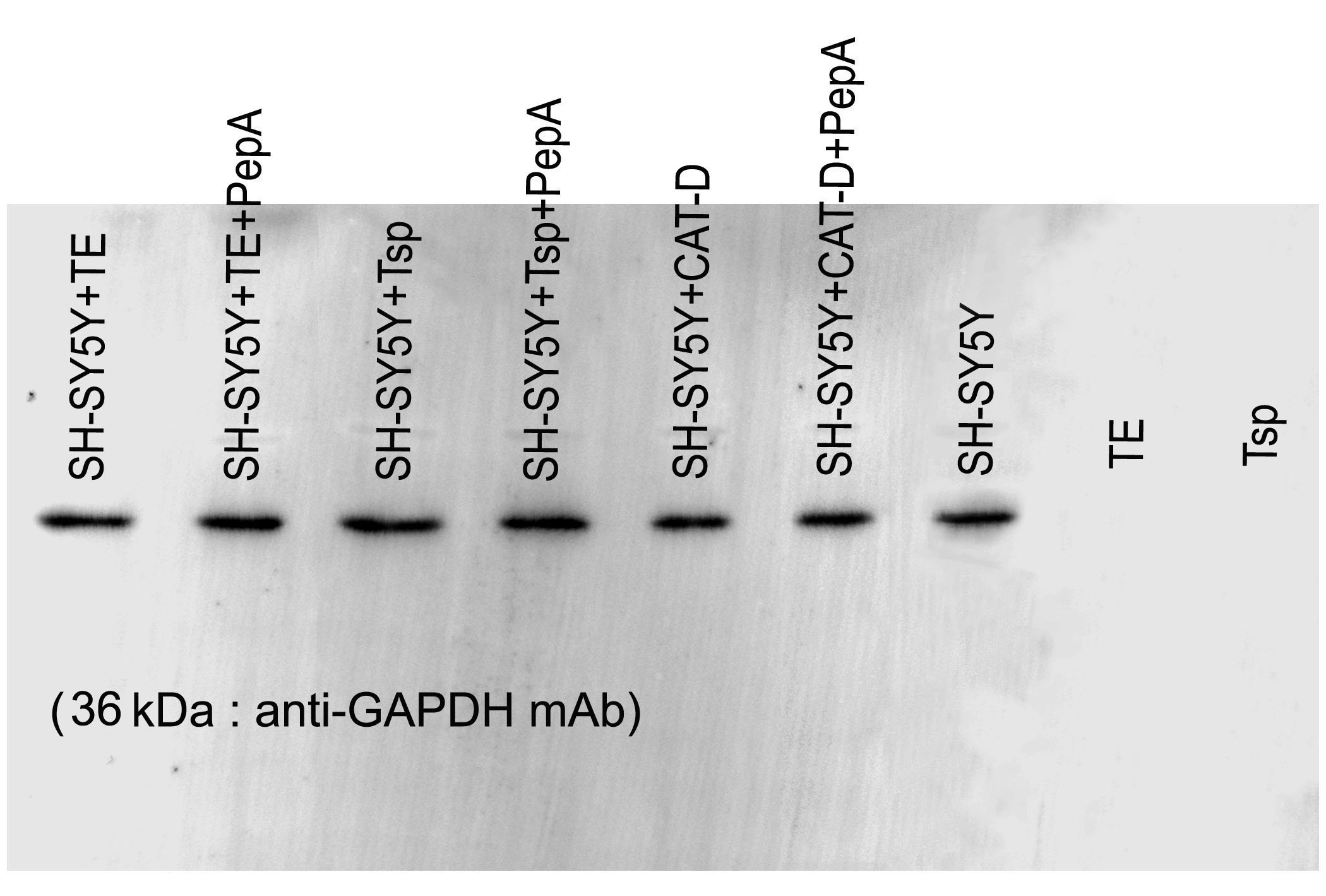

Supplement: Supplemental Information 9 [file peerj-08-10218-s009.jpg]
